# Development of a New Isoxsuprine Hydrochloride-Based Hydroxylated Compound with Potent Antioxidant and Anti-Inflammatory Activities

**DOI:** 10.4014/jmb.2405.05031

**Published:** 2024-10-03

**Authors:** Chien-Yu Wu, Hsiou-Yu Ding, Tzi-Yuan Wang, Chun-Wei Liu, Jiumn-Yih Wu, Te-Sheng Chang

**Affiliations:** 1 Department of Biological Sciences and Technology, National University of Tainan, Tainan 700301, Taiwan, Republic of China; 2 Department of Cosmetic Science, Chia Nan University of Pharmacy and Science, Tainan 717301, Taiwan, Republic of China; 3 Biodiversity Research Center, Academia Sinica, Taipei 115201, Taiwan, Republic of China; 4 Department of Food Science, National Quemoy University, Kinmen County 892009, Taiwan, Republic of China

**Keywords:** Anti-inflammation, antioxidant, hydroxylation, predicted data mining approach

## Abstract

The scientific community actively pursuits novel compounds with biological activities. In this context, our study utilized the predicted data mining approach (PDMA), which can efficiently screen out biotransformable precursor candidates to produce new bioactive compounds. The PDMA was applied to *Bacillus megaterium* tyrosinase (*Bm*TYR) to form new bioactive hydroxyl compounds from isoxsuprine hydrochloride (isoxsuprine). The results show that isoxsuprine could be biotransformed by *Bm*TYR to form a new compound, 3’’-hydroxyisoxsuprine. 3’’-Hydroxyisoxsuprine exhibited 40-fold and 10-fold higher potent antioxidant and anti-inflammation activities than the precursor, isoxsuprine. The 3’’-hydroxyisoxsuprine effectively mitigates the hyperimmune response in RAW 264.7 macrophages by inhibiting the upregulation of pro-inflammatory cytokine (IL-1β and IL-6) and inflammatory enzyme COX-2 gene expression triggered by LPS stimulation. This study illustrates that PDMA is an effective strategy for screening known natural and chemical compounds and for generating new bioactive compounds through biotransformation. Our newly produced compound has potential future applications in pharmacology and biotechnology.

## Introduction

The continual increase in the demand for new drugs for the treatment of a range of human diseases has led the scientific community to a significant and sustained interest in discovering new biologically active compounds. However, in organic chemistry and the pharmaceutical industry, synthesizing new compounds through chemical catalysis presents notable disadvantages such as high costs and the generation of environmentally harmful byproducts [1-3]. Recently, advancements in biochemical sciences have led to the discovery of numerous biocatalytic enzymes, enabling the sustainable production of new products under controlled and mild conditions [4, 5]. Enzymes can catalyze various reactions, including hydroxylation, glycosylation, hydrogenation, dehydrogenation, hydrolysis, *O*-methylation, and *O*-acetylation [6-10]. These biocatalytic processes offer a method for repurposing existing molecules with enantioselectivity and regioselectivity to facilitate the discovery of new compounds. This strategy is aimed at significantly reducing the reliance on nonselective, hazardous industrial chemicals and at minimizing waste generation to ultimately contribute to the production of value-added products [1-3, 10].

However, the biotransformation of known compounds into novel bioactive molecules represents a complex process, necessitating intensive labor and high costs for experimentation and optimization [11, 12]. To address this, in our previous studies, we developed the predicted data mining approach (PDMA), a rapid method for targeting new compounds via known enzymes and chemicals, which has been validated as an effective strategy [11]. The previous study focused on hydroxylation of tyrosine-like (phenolic) compounds using *Bacillus megaterium* tyrosinase (*Bm*TYR) to produce new catecholic products [11]. In fact, PDMA has high potential usefulness in different biotransformations using different types of enzymes. In previous studies, we successfully used tyrosinase to hydroxylate isoflavone, daidzin, genistein, glycitin, liquiritigenin, loureirin, and 2,3,5,4’-tetrahydroxystilbene-2-*O*-*β*-D-glucoside to produce *ortho*-hydroxylated flavonoids [12-16].

Hydroxylation, the process of converting a carbon-hydrogen bond to a carbon-hydroxyl bond, is one of the most widespread enzymatic activities [17]. This reaction represents one of the most common oxidative metabolic and functional group modification processes for a wide range of organic compounds, including beneficial pharmaceutical products, plant natural compounds, and harmful environmental pollutants [17, 18]. Hydroxylation has been shown to enhance the antioxidant activity of flavonoids [11, 14, 19], increase their ability to inhibit *α*-glucosidase or *α*-amylase activities [11, 20, 21], and boost their anti-inflammatory activities [22].

Through the implementation of the PDMA, strategies were established for database screening, which identified isoxsuprine hydrochloride (isoxsuprine) as a candidate. This compound can be hydroxylated by *Bm*TYR to yield novel compounds. Isoxsuprine [1-(p-hydroxyphenyl)-2-(1’-methyl-2’-phenoxyethylamino)-1-propanol hydrochloride], a derivative of epinephrine that belongs to the phenylethylamine class, was first synthesized by Moed and van Dijk in 1956 [23] to develop an orally administrable medication that could effectuate vasodilation while minimizing other cardiovascular effects, notably mitigating increased heart rate and reduced blood pressure [24, 25]. Isoxsuprine is described as a *β*-adrenoreceptor antagonist with *β*-adrenoreceptor-stimulating properties [26]. In humans, isoxsuprine is utilized for the treatment of peripheral arterial [27] and cerebrovascular insufficiency [26]. In addition, it is used as a tocolytic agent to inhibit preterm labor in both humans and animals [28]. However, as of now, no studies have investigated the use of isoxsuprine as a substrate for biotransformation to synthesize new compounds.

Herein, through screening using the PDMA, isoxsuprine was chosen as a substrate for biotransformation owing to its phenolic structure. We predicted that it can be hydroxylated by *Bm*TYR to form a catecholic product. In this study, we purified hydroxylated isoxsuprine products and characterized their structures. Furthermore, we identified the anti-inflammatory activities of the new substances through cellular assays and assessed their antioxidant activities using a 2,2-diphenyl-1-picrylhydrazyl (DPPH) assay.

## Materials and Methods

### Chemicals and Cells

The materials utilized in this study comprised the following: Murine macrophage RAW264.7 cells (BCRC 60001) were obtained from the Bioresources Collection and Research Center (BCRC) at the Food Industry Research and Development Institute, Hsinchu, Taiwan. These cells were cultured according to the BCRC protocols. Isoxsuprine was procured from Baoji Herbest Bio-Tech, Xi-An, Shaanxi, China. *Bacillus megaterium* tyrosinase (*Bm*TYR) with a specific activity of 4.84 U/mg was sourced from the authors of a prior study [15]. All additional reagents and solvents used were acquired commercially.

### Predicted Data Mining Approach (PDMA)

The strategy for precursor selection was based on a previous study [11]. In the pilot study, three criteria were set to survey the candidates, as follows: (1) to maximize the possibility of a reaction by *Bm*TYR, the structure of the compound candidate should contain a phenyl group that mimics the structure of tyrosine, the natural substrate of *Bm*TYR; (2) to obtain enough biotransformation products for further study, the candidate precursors should be available in industrial scale (gram-scale in this study); and (3) to produce a new product (*ortho*-dihydroxyl derivative) using *Bm*TYR, the predicted product should be novel in data banks. To satisfy criteria (1) and (2), suitable candidate precursors were selected from a catalog of commercially synthesized chemical molecules. The chemical structure of each predicted *ortho*-hydroxylation product from the biotransformation of the right candidate was drawn using the Reaxys program (RELX, UK), and the drawn compound was uploaded and searched in both the SciFinder databank (American Chemical Society, USA) and PubChem data banks to determine whether the predicted product was a new compound. The precursors with the predicted new derivatives were selected for function validation with *Bm*TYR.

### Biotransformation by *Bm*TYR

The biotransformation system was conducted with slight modifications from the method reported by Lee *et al*.[16]. The reaction mixture, consisting of 100 μl containing 500 mM borate (pH 9.0), 10 mM ascorbic acid, 1 mg/ml of the tested substrate compound (diluted from a 20 mg/ml stock in dimethyl sulfoxide [DMSO]), and 108 μg/ml *Bm*TYR, was incubated at 50°C with shaking at 200 rpm for 1.5 hours. To terminate the reaction, 20 μl of 1 M HCl and 120 μl of methanol (MeOH) were added to the mixture. Subsequent analysis was performed using high-performance liquid chromatography (HPLC).

### HPLC Analysis

The HPLC analysis was conducted according to methods described in a previous report [29]. The HPLC system used in this study was an Agilent 1100 series (Agilent, USA) equipped with a Waters 600 gradient pump (Waters, USA). The system was controlled via a PC workstation utilizing Chromatography Data Station software (SISC, Scientific Information Service Co., Ltd., Taiwan). The stationary phase incorporated a C18 column (5 μm, 4.6 i.d. × 250 mm; Sharpsil H-C18, Sharpsil, China). The mobile phase consisted of 1% acetic acid in water (A) and methanol (B). A linear gradient from 0 min with 20% B to 20 min with 50% B, an isocratic elution from 20 min to 25 min with 50% B, a linear gradient from 25 min with 50% B to 28 min with 20% B, and an isocratic elution from 28 min to 35 min with 20% B. All eluants were passed through the column at a flow rate of 1 ml/min. Each sample volume was set at 10 μl, and the detection condition was fixed at 280 nm.

To determine the chemical structure of the products, the biotransformation reaction was upscaled up to 20 ml (1 ml per tube) for *Bm*TYR biotransformation. The metabolites were purified using the preparative YoungLin HPLC system. After the completion of the reaction, the metabolites were purified using a preparative YoungLin HPLC system (YL9100, YL Instrument, Republic of Korea). The elution corresponding to the metabolite peaks in the HPLC analysis was collected, concentrated by a rotary evaporator (Rotavapor R-100, Bunchi Co., Japan), and then dehydrated using a freeze dryer (Kingmech Sci. Co., Ltd., Taiwan). The structures of the compounds were verified using nucleic magnetic resonance (NMR) and mass spectral analyses, which were carried out with a mass spectrometer (AB Sciex Instruments QTRAP 5500; Applied Biosystem Corp., USA) equipped with electrospray ionization. The mass spectrometry parameters included a capillary voltage ranging from 4.5 to 5.5 kV and a desolvation gas pressure of 20 psi. Mass signals were collected using a single-ion recording method and processed using the Analyst 1.5 software (Applied Biosystem Corp.). Full mass and mass-mass scan data were acquired with a mass range of 100 to 1,150 *m/z* in the positive ion mode. For the NMR analysis, a 5-mm-diameter NMR tube containing 5 mg of the purified product compound dissolved in 0.5 ml of DMSO-d6 was prepared. Subsequently, ^1^H- and ^13^C-NMR, distortionless enhancement polarization transfer (DEPT), heteronuclear single quantum coherence (HSQC), heteronuclear multiple-bond connectivity (HMBC), correlation spectroscopy (COSY), and nuclear Overhauser effect spectroscopy (NOESY) were recorded using a high-resolution Bruker AV-700 NMR spectrometer (Bruker Co., USA) at an ambient temperature. Standard pulse sequences and parameters were used for the NMR experiments, and all chemical shifts were reported in parts per million (ppm, δ).

### Determination of Anti-Oxidant Activity

The assay was performed as previously described, with minor modifications [15]. The test sample, dissolved in dimethyl sulfoxide (DMSO), was combined with a DPPH solution (1 mM in methanol) to a final volume of 0.1 ml. After a reaction time of 15 minutes, the absorbance of the reaction mixture was measured at 520 nm using a microplate reader (Sunrise, Tecan, Switzerland). Ascorbic acid, also dissolved in DMSO, served as a positive antioxidant standard. The DPPH free radical scavenging activity was calculated using the formula: (OD_520_ of the control reaction - OD_520_ of the reaction)/(OD_520_ of the control reaction). The concentration at which 50% of the initial DPPH radicals were scavenged under the assay conditions was defined as the half-maximal inhibitory concentration (IC_50_).

### Cell Culture and Sample Treatment

The murine macrophage RAW 264.7 cell line was cultured in Dulbecco's Modified Eagle Medium (DMEM) supplemented with 10% fetal bovine serum, 100 μg/l streptomycin, and 100-IU/ml penicillin, maintained at 37°C in a 5% CO_2_ atmosphere. Cells were seeded in 6-well / 24-well plates and incubated for 24 h at 37°C and 5% CO_2_. Various concentrations of the tested compounds were added. After 1 h of treatment, cells were stimulated with 6.25 ng/ml lipopolysaccharide (LPS) for 24 h. Various concentrations of test compounds dissolved in DMSO. Cells were treated with 0.05% DMSO as vehicle control.

### Determination of Anti-Inflammatory Activity

Anti-inflammatory activity was evaluated by monitoring the nitric oxide (NO) production in lipopolysaccharide (LPS)-induced macrophage cells [29]. Cells were seeded at a density of 5 × 10^5^ cells/well in 24-well plates and incubated for 24 h at 37°C and 5% CO_2_. Various concentrations of the tested compounds were added. After 1 hour of treatment, cells were stimulated with 6.25 ng/ml lipopolysaccharide (LPS) for 24 h. Nitric oxide (NO) production was assessed by mixing equal volumes of culture supernatants with Griess reagent at room temperature for 10 minutes; absorbance was measured at a wavelength of 540 nm using a microplate reader (Sunrise, Tecan). 3-(4,5-Dimethylthiazol-2-yl)-2,5-diphenyl tetrazolium bromide (MTT) assays were performed to determine the cytotoxicity of the compounds tested on RAW cells. The culture medium was removed for the described Griess assay, replaced with a 1-mg/ml MTT solution dissolved in phosphate-buffered saline, and incubated for an additional 2 h. The MTT solution was then removed, and DMSO was added, after which the absorbance of the dissolved formazan crystals was determined at 570 nm with a microplate reader (Sunrise, Tecan). These analyses were performed in triplicate. The relative inhibition of NO production was calculated with the following equation: Relative inhibition (%) = [(OD_540_ with LPS only - OD_540_ with both LPS and the tested compounds)/(OD_540_ with LPS only - OD_540_ without LPS or isoflavone)] × 100%. An IC_50_ value indicates a drug concentration that exhibits 50% inhibition. Relative cell survival was calculated using OD_570_ without LPS or isoflavone normalized as 100%.

### Total RNA Extraction

Cells were seeded at a density of 3 × 10^6^ cells/well in 6-well plates and incubated for 24 h at 37°C and 5% CO_2_. Various concentrations of the tested compounds were added (140 μM or 35 μM). After 1 h of treatment, cells were stimulated with 6.25 ng/ml lipopolysaccharide (LPS) for 24 h. After conducting the treatment experiments, three tilapia larvae were collected as a sample, and three replicates (*n* = 3) were performed. Total RNA was extracted from collected samples using Trizol reagent (Thermo Fisher Scientific, USA) as per the manufacturer’s instructions. Briefly, the samples were homogenized in 1 ml Trizol reagent, and 0.2 ml chloroform was added and thoroughly mixed by shaking. The mixture was then centrifuged at 12,000 × g at 4°C for 45 min. An equal volume of isopropanol was added to the sample, and the mixture was centrifuged at 12,000 ×*g* at 4°C for 30 min to precipitate total RNA pellets. The pellets were washed twice with 70% alcohol for 30 min at 12,000 × g at 4°C. The quantity and quality of total RNA were assessed using absorbance at 260 and 280 nm, as well as gel electrophoresis, and then stored at -20°C until further use.

### Real-Time PCR

The experimental protocols were adapted from previous studies with minor modifications [30]. The cDNA of RAW264.7 samples was synthesized using the GoScript Reverse Transcription System (Promega, USA) and poly(dT) primers. The mRNA used for cDNA synthesis was treated with DNase I (Promega) to eliminate genomic DNA contamination. Real-time PCR was carried out using a StepOne Plus Real-Time PCR System (Applied Biosystems, Thermo Fisher Scientific, USA) in a final reaction volume of 20 μl, consisting of 10 μl Fast SYBR Green Master Mix (Applied Biosystems, Thermo Fisher Scientific), 400 nM forward and reverse primers, 15 ng cDNA, and nuclease-free water. The sequences of the primers used are as follows: Cyclooxygenase-2 (COX-2) forward: GCGACATACTCAAGCAGGAGCA, reverse: AGTGGTAACCGCTCAGGTGTTG; interleukin(*IL*)-*1β* forward: CCTGGGCTGTCCTGATGAGAG, reverse: TCCACGGGAAAGACACAGGTA; *IL-6* forward: AAGTGCATCATCGTTGTTCATACA, reverse: GAGGATACCACTCCCAACAGACC; GAPDH forward: CAATGTGTCCGTCGTGGATCT, reverse: GTCCTCAGTGTAGCCCAAGATG. All primer sequences used in the experiments were sourced from previous studies [30-32]. The GAPDH was used as an internal control to normalize mRNA expression and was taken from previous studies [31].

### Statistical Analysis

Statistical analysis was done using GraphPad Prism (GraphPad Inc., USA). The normality of the data was tested with the Shapiro-Wilk normality test. All data presented in this study represent the averages of the values from the experiments conducted in triplicate (*n* = 3) and are expressed as mean ± standard deviation (SD). A statistical analysis was performed using the Student’s *t*-test. A *p*-value of < 0.05 was deemed statistically significant.

## Results

### PDMA to Seek Novel Biotransformable Derivatives

**Fig. 1:**
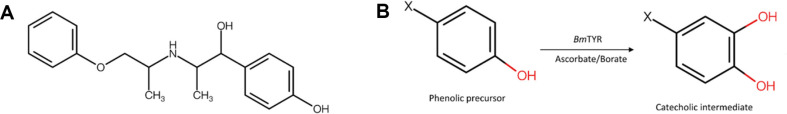
Design of biotransformation strategies and screening of precursor compound using PDMA. (**A**) Chemical structure of precursor, isoxsuprine. (**B**) Enzymatic cascade biotransformation using *Bm*TYR.

Previous studies have shown that *Bm*TYR is an appropriate biocatalyst not only in the *ortho*-hydroxylation of flavonoids [13-16, 33] but also in other biotransformations [34]. We hypothesized that *Bm*TYR can biotransform precursor compounds with phenolic groups that mimic the structure of tyrosine, resulting in the generation of catechol products (Fig. 1B). This process is applicable not only to natural compounds but also to chemically synthesized compounds. Using the PDMA we developed in our previous study [11], we expanded our screening targets to include commercially synthesized chemical compounds. Among these compounds, isoxsuprine, a vasodilator used in humans, was chosen as a candidate precursor and was expected to be hydroxylated into new compounds using *Bm*TYR (Fig. 1A).

### New Biotransformable Derivatives from Enzymatic Synthesis of Isoxsuprine Hydrochloride

**Fig. 2:**
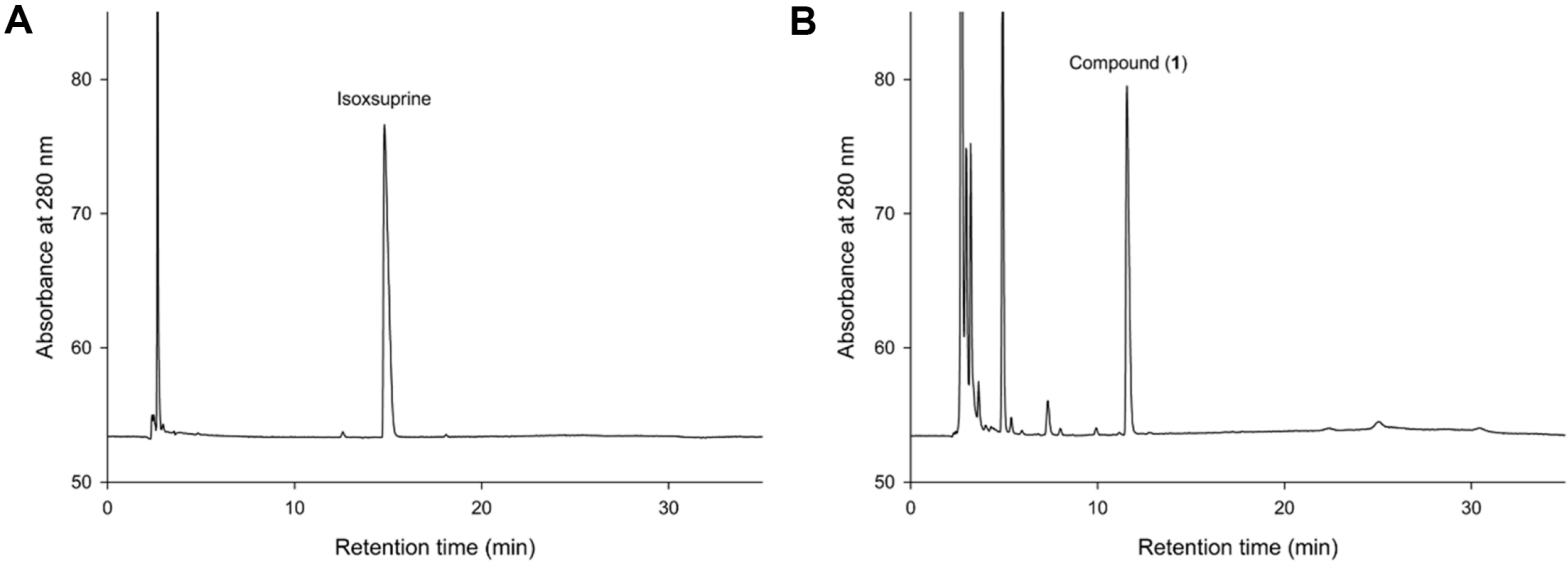
High-performance liquid chromatography (HPLC) analysis of the biotransformation products of isoxsuprine using *Bm*TYR (A, B). The biotransformation is described in the materials and methods part of the paper. At the end of the reaction, the reaction mixture was analyzed using HPLC, as described in the materials and methods part of the paper.

Isoxsuprine was identified from a commercial compound catalog using the PDMA and subjected to biotransformation by *Bm*TYR. The results demonstrated that isoxsuprine was successfully biotransformed by *Bm*TYR, resulting in a product compound (Fig. 2A and 2B). To determine the chemical structure of the product compound, we performed a scale-up of the biotransformation process, followed by purification of the product and structural identification.

**Fig. 3:**
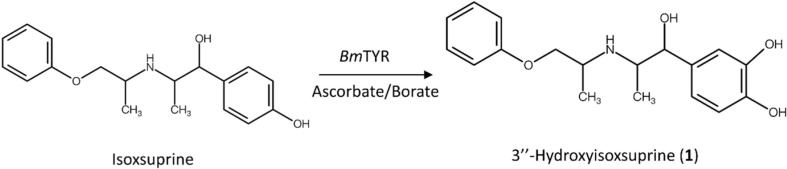
Biotransformation of isoxsuprine hydrochloride using the *Bm*TYR reaction.

The chemical structure of the purified compound (**1**) was then analyzed using high-resolution mass spectrometry (HRMS) and NMR spectral analyses. The molecular formula of compound (**1**) was established as C_18_H_24_O_4_N by HRMS at *m/z* 318.1700 [M+H]^+^ (Fig. S1). In addition, this study presents a broad conformation of complementary one-dimensional (1D) and 2D NMR techniques. The ^1^H-NMR spectra (500 MHz, CD_3_OD) provided the following chemical shifts (δ_H_): 1.15 (3H, d, *J* = 6.5 Hz, H-3), 1.52 (3H, d, *J* = 6.5 Hz, H-3’), 3.61 (1H, dd, *J* = 3.0, 6.5 Hz, H-2), 3.93 (1H, m, H-2’), 4.20 (1H, dd, *J* = 5.8, 10.6 Hz, H-1a’), 4.34 (1H, dd, *J* = 3.0, 10.6 Hz, H- 1b’), 5.08 (1H, d, *J* = 3.0 Hz, H-1), 6.70 (1H, dd, *J* = 1.8, 8.0 Hz, H-6’’), 6.78 (1H, d, *J* = 8.0 Hz, H-5’’), 6.82 (1H, d, *J* = 1.8 Hz, H-2’’), 7.02 (1H, t, *J* = 7.5 Hz, H-4’’’), 7.03 (2H, d, *J* = 7.5 Hz, H-2’’’, 6’’’), and 7.34 (2H, t, *J* = 7.5 Hz, H-3’’’, 5’’’). The results observed in the ^13^C-NMR spectra (125 MHz, CD_3_OD) were as follows: δ_C_ 11.1 (C-3), 15.0 (C-3’), 52.6 (C-2’), 58.9 (C-2), 69.4 (C-1’), 71.3 (C-1), 114.3 (C-2’’), 115.9 (C-3’’’, 5’’’), 116.5 (C-5’’), 118.4 (C-6’’), 123.1 (C-4’’’), 130.9 (C-2’’’, 6’’’), 133.0 (C-1’’), 146.3 (C-4’’), 146.8 (C-3’’), and 159.4 (C-1’’’). The resonance bands in the chemical shift range of δ_H_ 4.20 (dd, *J* = 5.8, 10.6 Hz, H-1a’) and 4.34 (dd, *J* = 3.0, 10.6 Hz, H-1b’) exist as doublets of doublets correlated to the methylene group at position 1’. This evidence to assign H-1’ and H-2’ was obtained from the homonuclear COSY spectrum. The doublet at a chemical shift of 5.08 ppm was assigned to the benzylic methine at position 1 (d, *J* = 3.0 Hz), and the multiplet at 3.61 ppm previously assigned to CH at position 2 confirmed this assignment. On the basis of the chemical shift assignments and coupling constants, the two methine functions at positions 2 and 2’ could be correlated to the two multiplets at chemical shifts of 3.61 and 3.93. ppm. The ^1^H-NMR shows two doublet absorptions at chemical shifts of 1.15 and 1.52 ppm (*J* = 6.5 Hz), which could be correlated to the two methyl groups at positions 3 and 3’. Of the eight aromatic protons, the remaining three appeared as an ABX pattern at 6.78 (1H, d, *J* = 8.0 Hz), 6.70 (1H, dd, *J* = 1.8, 8.0 Hz), and 6.82 (1H, d, *J* = 1.8 Hz), assigned to H-5’’, H-6’’, and H-2’’, respectively, representing the 1,3,4-trisubstituted aromatic pattern of the polyphenol residue. The three multiplets at 7.02 (1H, t, *J* = 7.5 Hz, H-4’’’), 7.03 (2H, d, *J* = 7.5 Hz, H-2’’’, 6’’’), and 7.34 (2H, t, *J* = 7.5 Hz, H-3’’’, 5’’’), assigned to H-4’’’, H-2’’’, H-6’’’, H-3’’’, and H-5’’’, were integrated for 3 and 2 protons, and correlated to the phenoxy moiety. The full assignments of the ^1^H and ^13^C-NMR signals were further aided with DEPT, COSY, HMBC, HSQC, and NOESY spectra. Figs. S2–S8 demonstrate the structure of compound (**1**) to be 3’’-hydroxyisoxsuprine (**1**) (Fig. 3). The structure of the product of hydroxylated isoxsuprine was identified as 3’’-hydroxyisoxsuprine. As expected, 3’’-hydroxyisoxsuprine is a new compound.

### 3’’-Hydroxyisoxsuprine Hydrochloride Possesses Potent Anti-Oxidant Activity

**Fig. 4:**
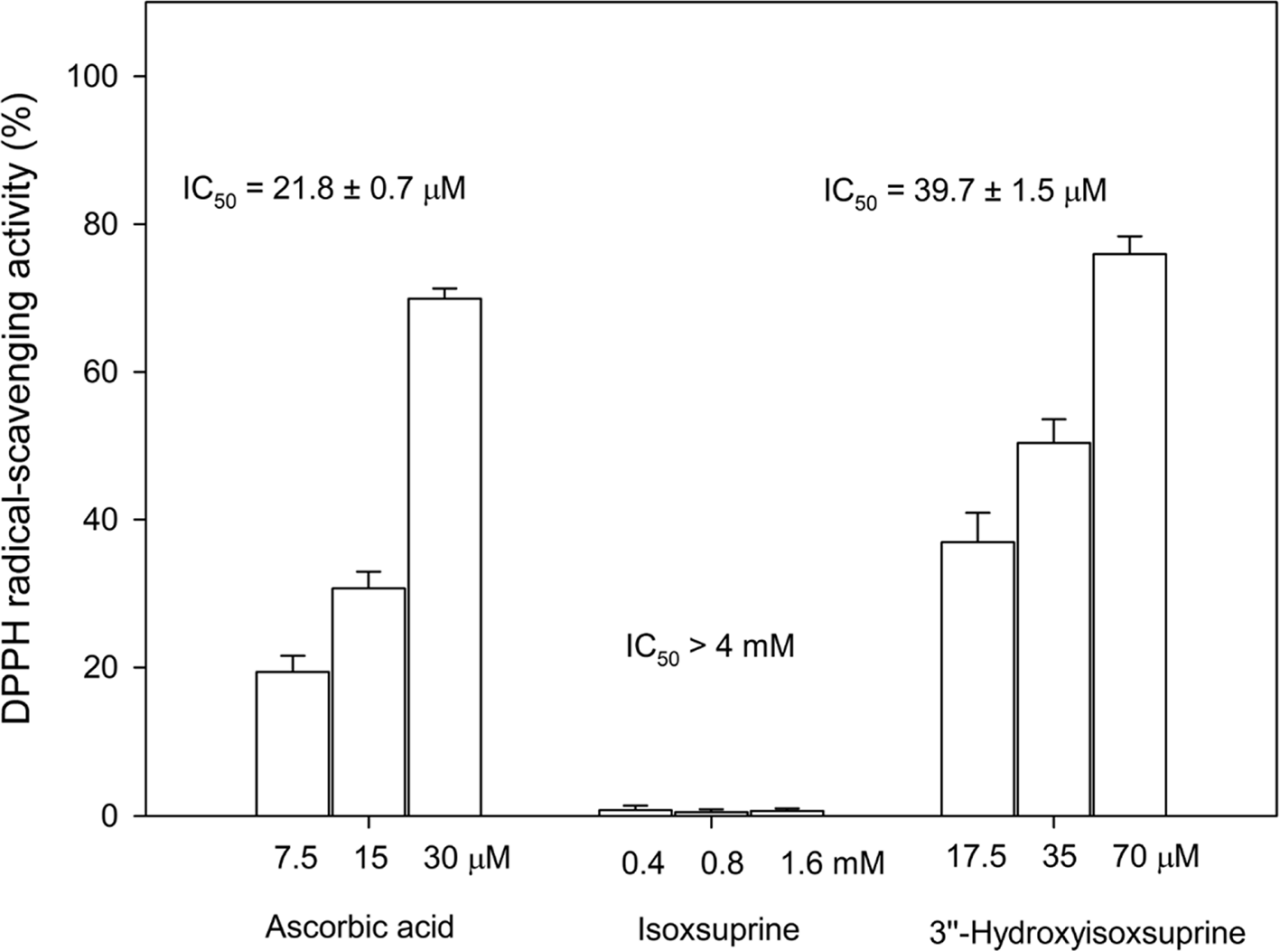
DPPH free radical-scavenging activity of ascorbic acid, isoxsuprine, and its derivative, 3’’-hydroxyisosuprine. The DPPH scavenging activity was determined as described in the materials and methods part of the paper. The mean (*n* = 3) is shown, and the S.D. are represented by the error bars. The IC_50_ values represent the concentrations required to inhibit the DPPH free radical-scavenging activity by 50%.

Studies have reported that the *ortho*-dihydroxyl groups on the benzene ring of flavonoid structures are crucial in mediating their antioxidant activity [10, 19, 35]. Thus, the antioxidative activities of both 3’’-hydroxyisoxsuprine and its precursor isoxsuprine were determined using the DPPH free radical scavenging assay. To compare the antioxidant activity of isoxsuprine and its derivative, the compounds were evaluated for DPPH free radical scavenging activity in serious concentrations. The results showed that 3’’-hydroxyisoxsuprine had a potent antioxidant activity with an IC_50_ of 39.7 ± 1.5 μM, which was 40-fold higher than that of isoxsuprine (Fig. 4).

### 3’’-Hydroxyisoxsuprine Hydrochloride Possess Potent Anti-NO Activity

**Fig. 5:**
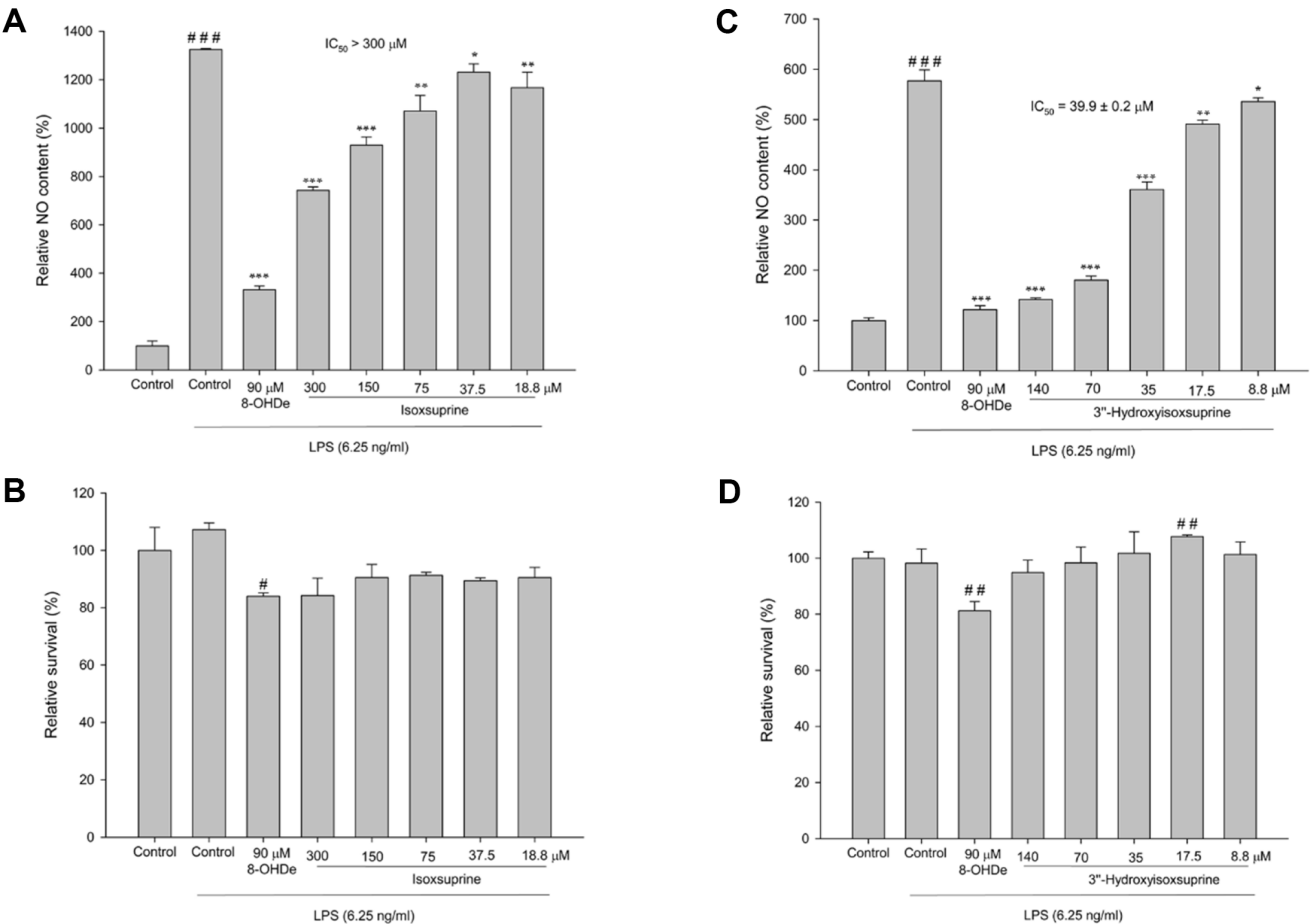
Effects of isoxsuprine (A, B) and 3’’-hydroxyisosuprine (C, D) on the inhibition of lipopolysaccharide (LPS)-induced nitric oxide (NO) production (A, C) and cell survival (B, D) in murine macrophage RAW264.7 cells. The cells were incubated with isoflavone at the indicated concentrations for 1 h before treatment with LPS (6.25 ng/ml) for 24 h. The amounts of NO were determined using Griess reagent in the culture medium. Cell viability was determined with an MTT assay. Each value indicates the mean ± standard deviation (SD) and is representative of the results obtained from three independent experiments. ^#^*p* < 0.05, ^##^*p* < 0.01 ^###^*p* < 0.001 versus Control group; *p < 0.05, **p < 0.01, ***p < 0.001 versus Control group treated with LPS.

Research has highlighted that the catechol structures of compounds significantly contributes to their anti-inflammatory activities [22, 36-38]. To compare the anti-NO activity of isoxsuprine and its derivative, the compounds were evaluated for anti-NO activity in serious concentrations. The results showed that 3’’-hydroxyisoxsuprine had a potent anti-NO activity with an IC_50_ of 39.9 ± 0.2 μM, which was sevenfold higher than that of isoxsuprine (Fig. 5).

### 3’’-Hydroxyisoxsuprine Hydrochloride Suppresses LPS-Induced *COX-2*, *IL-1β* and *IL-6* mRNA Expression in RAW 264.7

**Fig. 6:**
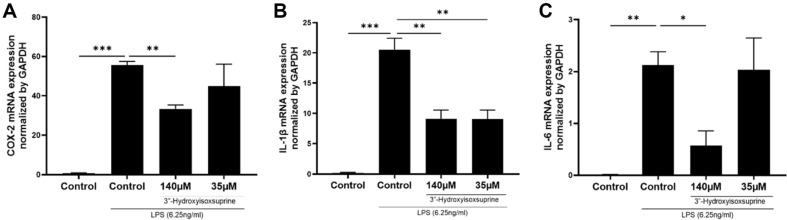
Effects of 3’’-hydroxyisosuprine on lipopolysaccharide (LPS)-induced mRNA expression of COX-2 (A), IL-1β (B) and IL-6 (C) in RAW 264.7 cells. The cells were not treated or were pretreated with 3’’-hydroxyisosuprine (35 or 140 μM) for 1 h prior to being stimulated with LPS (6.25 ng/ml) for 24 h. Total RNA was prepared from cells pretreated with or without the indicated concentrations of 3’’-hydroxyisosuprine for 2 h and then stimulated with LPS (6.25 ng/ml) for 24 h. The mRNA levels of *COX-2*, *IL-1β* and *IL-6* were determined by real-time PCR and the values were normalized to GAPDH. Each value indicates the mean ± standard deviation (SD) and is representative of the results obtained from three independent experiments. *p < 0.05, **p < 0.01 and ***p < 0.001 statistically significantly different from the value for the cells treated with LPS.

Given the significant efficacy of 3’’-hydroxyisoxsuprine in anti-NO activity, we further explored its role in modulating anti-inflammatory responses. LPS triggers the secretion of a variety of pro-inflammatory cytokines such as IL-1β and IL-6 and inflammatory enzyme COX-2 [30-32, 39, 40]. We assessed the effects of 3’’-hydroxyisoxsuprine on the upregulation of *COX-2*, *IL-1β*, and *IL-6* mRNA expression in LPS-induced RAW 264.7 macrophages using real-time PCR. LPS stimulation significantly increased the mRNA expression of *COX-2*, *IL-1β*, and *IL-6*, while the basal levels of these cytokines were typically observed in macrophages. We also examined whether 3’’-hydroxyisoxsuprine could attenuate the increase in pro-inflammatory cytokines in LPS-induced RAW 264.7 macrophages (Fig. 6). Pre-treatment with 3’’-hydroxyisoxsuprine downregulated *COX-2* (Fig. 6A), *IL-1β* (Fig. 6B), and *IL-6* (Fig. 6C) mRNA expression. The results indicate that 3’’-hydroxyisoxsuprine effectively mitigates the hyperimmune response in RAW 264.7 macrophages by inhibiting the upregulation of pro-inflammatory cytokine (IL-1β and IL-6) and inflammatory enzyme COX-2 gene expression triggered by LPS stimulation. Therefore, the novel compound 3’’-hydroxyisoxsuprine, produced through biotransformation, indeed exhibits anti-inflammatory activity.

## Discussion

In this study, we used the PDMA to discover new compounds originating from isoxsuprine. Our team abandoned the conventional screening method that randomly pairs enzymes with various precursors, instead adopting a more successful strategy focused on specific enzymes. We designated *Bm*TYR as the core enzyme for this PDMA screening. Through the PDMA strategy, we substantially enhanced the efficiency of synthesizing new compounds via biotransformation, while also reducing the high costs and resource wastage associated with previous random screening methods. Utilizing a software platform, we screened for and predicted novel compounds capable of biotransformation into catechol structures that have not yet been reported in the literature (not found in SciFinder databank and PubChem data bank). Through the PDMA process, a new isoxsuprine derivative was identified. The bioactivities of the products were then confirmed.

Fig. 3 shows the enzymatic biotransformation of isoxsuprine by *Bm*TYR. This finding indicates that isoxsuprine was biotransfomed by *Bm*TYR to form a catecholic product, 3’’-hydroxyisoxsuprine. In this study, the hydroxy product was confirmed as a novel compound, thus supporting the efficacy of the PDMA strategy in successfully identifying new biotransformable derivatives from among hundreds of available compounds. The PDMA has value in predicting candidates for the hydroxylation of natural compounds using *Bm*TYR[11] and the glycosylation of natural compounds using bacterial GTs (submitted).

In this study, we evaluated the bioactivities of compounds produced through biotransformation that introduce *ortho*-dihydroxyl groups. Previous studies have indicated that *ortho*-dihydroxyl groups on the benzene ring of flavonoid structures are critical for mediating their antioxidant activities [10, 19, 35]. Research has also highlighted that the catechol structure of compounds significantly contributes to their anti-inflammatory activities [22, 36-38]. Our results demonstrate that 3’’-hydroxyisoxsuprine effectively downregulated the gene expression of inflammatory factors induced by LPS, including pro-inflammatory cytokines such as IL-1β and IL-6 and inflammatory enzyme COX-2 (Fig. 6).

The antioxidant and anti-inflammatory properties of natural products have been a prominent research topic in recent years. Numerous studies have indicated that natural compounds containing catechol structures possess antioxidant and anti-inflammatory functions [39-41]. The effect could be related to the known effects of lowering the level of ROS and ameliorating mitochondrial dysfunction [41]. In this study, we confirmed that 3’’-hydroxyisoxsuprine downregulates the gene expression of pro-inflammatory cytokines (IL-1β and IL-6) and inflammation-related enzymes (COX-2) induced by LPS (Fig. 6). However, the mechanisms of antioxidant and anti-inflammatory actions are complex, and the detailed mechanism of 3’’-hydroxyisoxsuprine requires further investigation.

The chemical structure of the newly synthesized compound, 3’’-hydroxyisoxsuprine, aligns with PDMA predictions and demonstrates both antioxidant (Fig. 4) and anti-inflammatory bioactivities (Fig. 5). Thus, the PDMA can indeed be used to discover new bioactive compounds with various enzymes and other commercially available natural or chemically synthesized compounds. Therefore, future studies on the biotransformation of diverse compounds using the PDMA with different types of enzymes are warranted. Furthermore, in future research, we plan to further optimize the PDMA process by incorporating software-assisted molecular docking simulations [42, 43]. This simulation approach will predict the efficiency of biotransformation and the bioactivity of the transformed compounds, thereby enhancing the efficiency of generating new bioactive compounds. This refined process is expected to reduce human and resource consumptions in experimental procedures and to further minimize waste production.

Currently, environmental regulations, competitiveness, and social responsibility have compelled industries to develop more environment-friendly and cost-effective processes for the production of industrially important compounds [44]. For these reasons, an increasing number of researchers have engaged in the development of biotransformation technologies. By utilizing enzymes naturally derived from microbes, plants, and animals, these processes have achieved high efficiency and specificity in chemical reactions [45]. Moreover, such technologies offer significant advantages in terms of resource efficiency, cost-effectiveness, energy conservation, and environmental friendliness [45]. In this study, the PDMA was demonstrated to be effective in discovering new bioactive compounds, aligning with the current trend toward green chemistry in the chemical industry. This method holds potential for the future exploration of compounds with pharmaceutical properties.

The compound isoxsuprine has a long history of being synthesized and utilized as a pharmaceutical agent [23]. In this study, we successfully generated a new bioactive compound through the biotransformation of isoxsuprine. The new compound was successfully identified as 3’’-hydroxyisoxsuprine, which exhibited a bioactivity that was not observed in the original isoxsuprine. The new compound exhibited strong antioxidant and anti-inflammatory activities. This study successfully endowed an old drug with new pharmacological value through biotransformation. The new compound may have the potential to be developed as a multifunctional drug. Further research into the pharmacological mechanisms of this new compound is warranted.

## Conclusion

In conclusion, this study proves that the PDMA is a validated approach for synthesizing new bioactive compounds from known substances. We can strategically design functional group additions to anticipate the potential bioactivity of new compounds, select appropriate enzymes, and then screen for compounds that may undergo biotransformation with functional group additions by these enzymes. The study results confirm that in addition to natural compounds, chemically synthesized compounds can also be transformed into new bioactive compounds through this strategy. The newly synthesized compound, 3’’-hydroxyisoxsuprine, exhibits antioxidant and anti-inflammatory bioactivities. Preliminary findings suggest that the 3’’-hydroxyisoxsuprine effectively mitigates the hyperimmune response in RAW 264.7 macrophages by inhibiting the upregulation of pro-inflammatory cytokine (IL-1β and IL-6) and inflammatory enzyme COX-2 gene expression triggered by LPS stimulation. Future studies are necessary to further investigate the pharmacological properties and value of this compound, and to assess the feasibility of this biotransformation process for commercial applications.

## Supplemental Materials

Supplementary data for this paper are available on-line only at http://jmb.or.kr.
