# Supplementary material for: Development of a New Isoxsuprine Hydrochloride-Based Hydroxylated Compound with Potent Antioxidant and Anti-Inflammatory Activities
Source: J Microbiol Biotechnol. 2024 Oct 3;34(12):2693–701. doi: 10.4014/jmb.2405.05031 (PMC11729693; doi:10.4014/jmb.2405.05031)
Supplement: Supplementary file 1 [file jmb-34-12-2693-supple.pdf]

J. Microbiol. Biotechnol. 2024. 34(12): 2693–2701
https://doi.org/10.4014/jmb.2405.05031

# Supplementary Figures

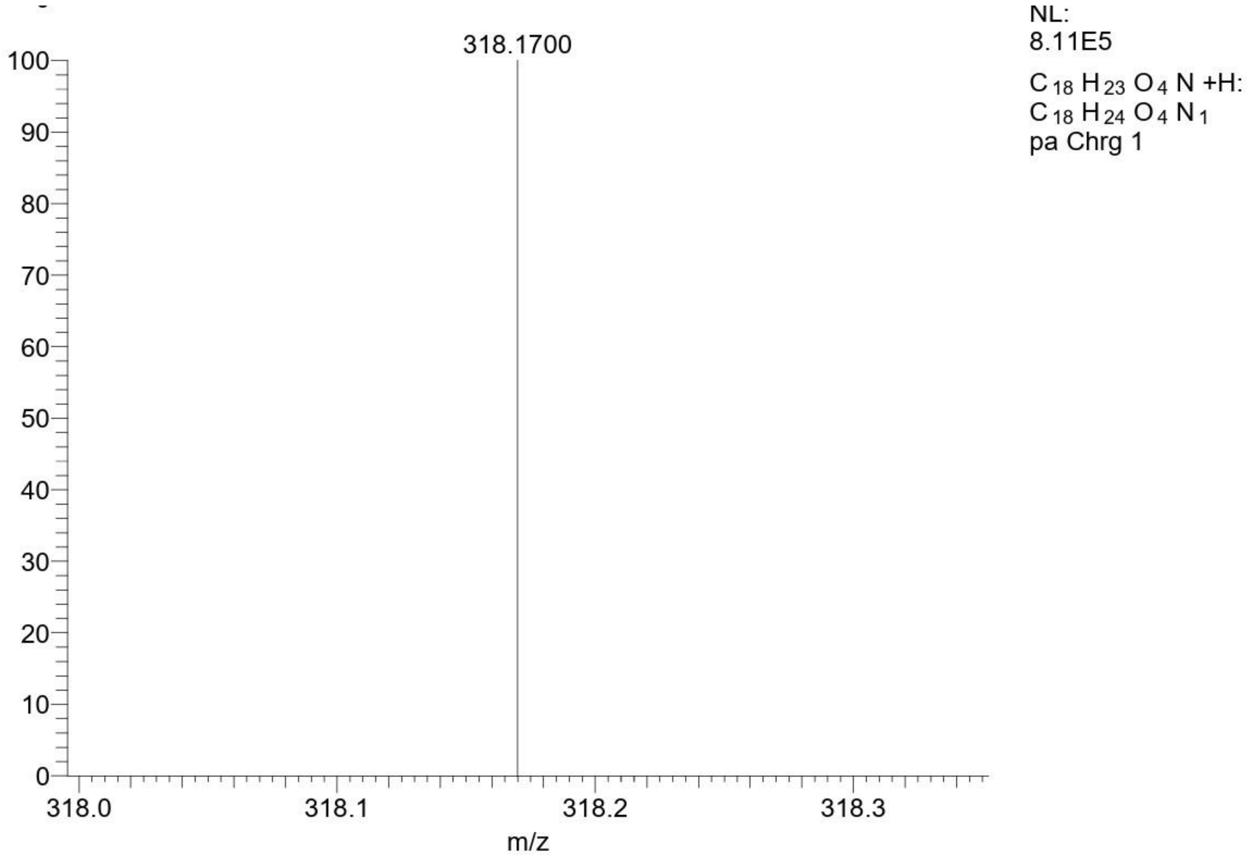


**Fig. S1. High-resolution mass spectrometry analysis of 3″-hydroxyisosuprine (1) in the positive mode. A significant signal can be observed at 318.1700 *m*/*z*.**

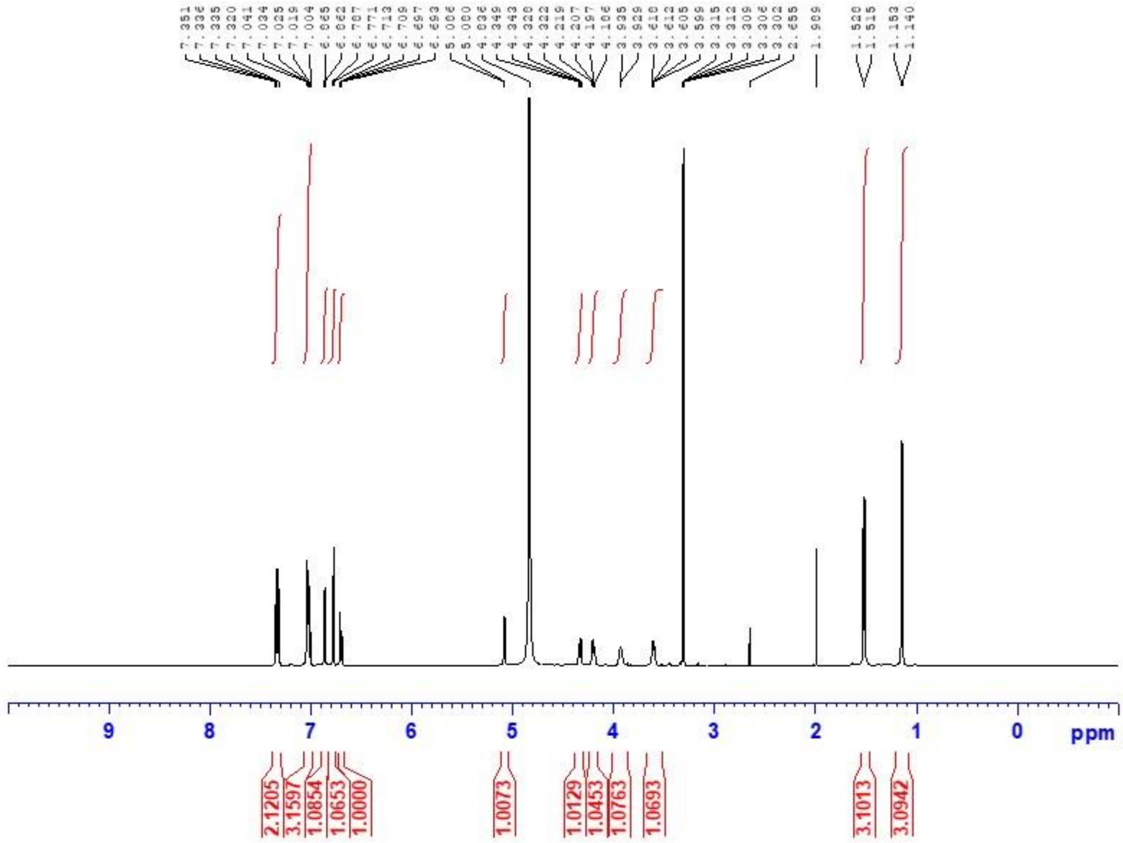

**Fig. S3. 1D NMR spectrum ($^1$H-NMR, 500 MHz, $CD_3OD$) of 3″-hydroxyisosuprine (1).**

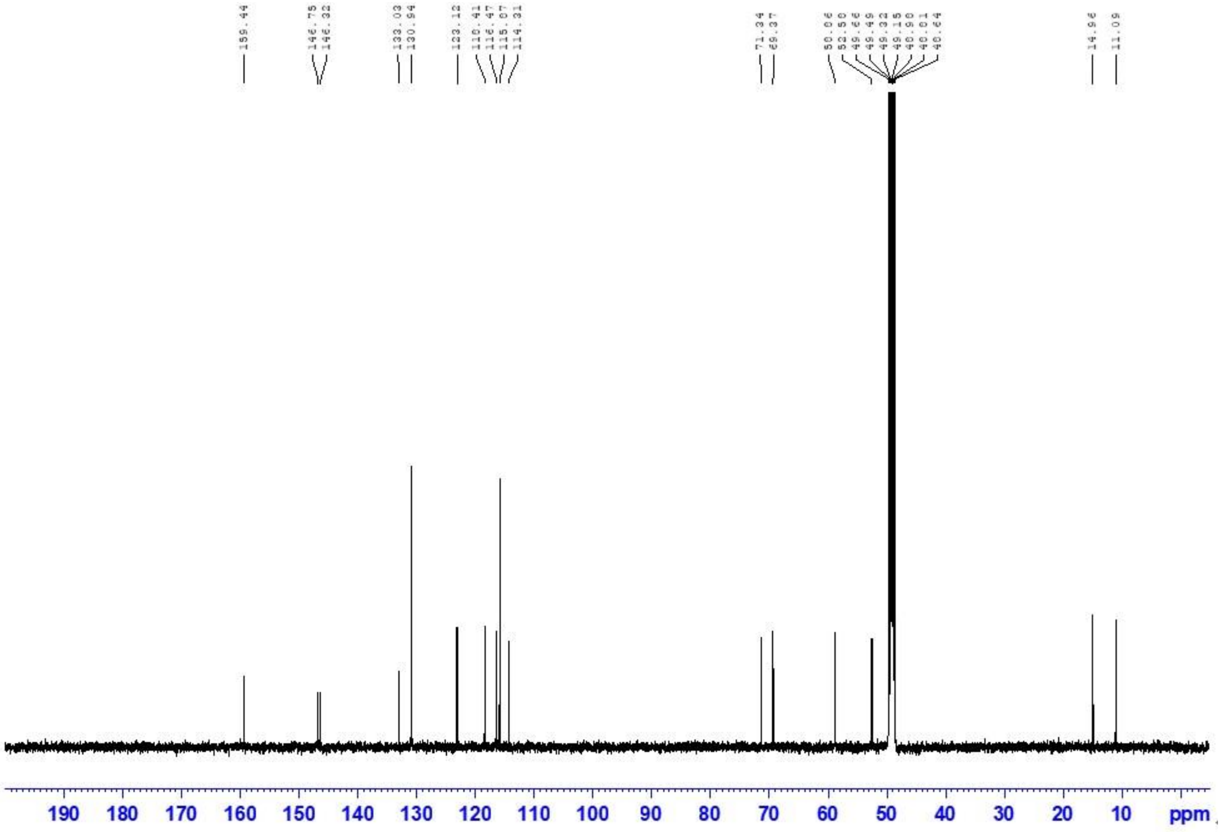


**Fig. S4. 1D NMR spectrum ($^{13}$C-NMR, 125 MHz, $CD_3OD$) of 3″-hydroxyisosuprine (1).**

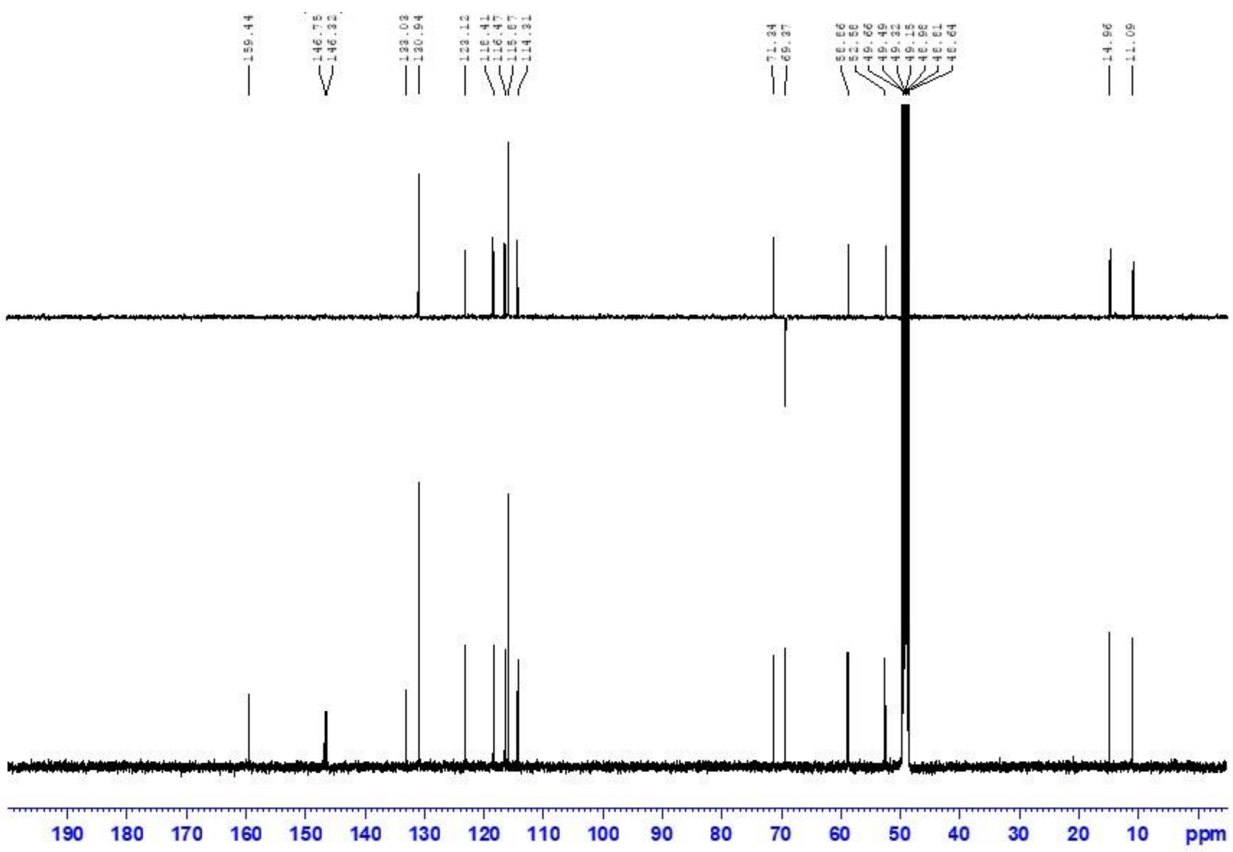


**Fig. S5. 1D NMR spectrum (DEPT-135, 125 MHz, $CD_3OD$) of 3″-hydroxyisosuprine (1).**

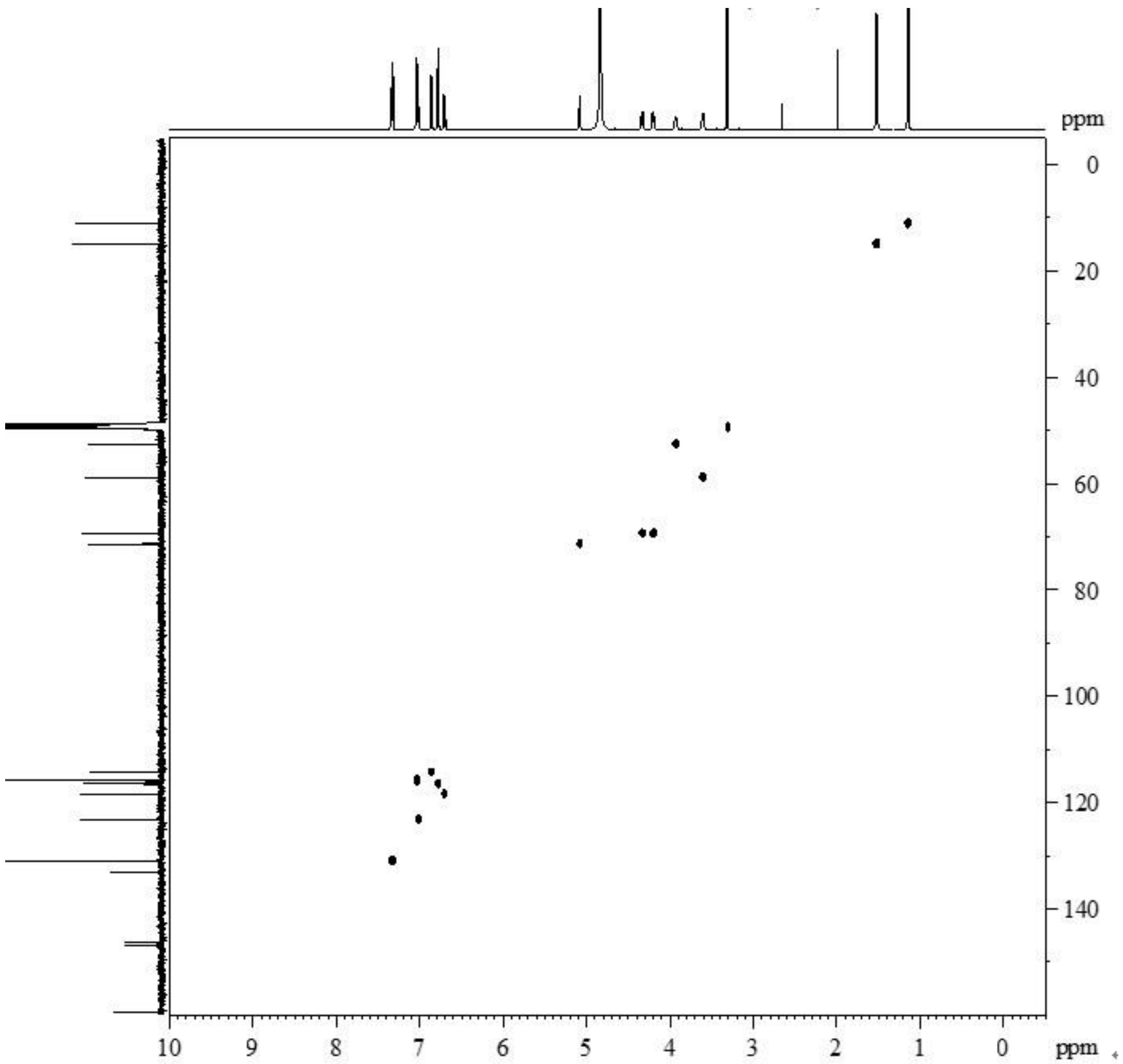


**Fig. S6. 2D NMR spectrum ($^1H$-$^{13}C$ HSQC, 500 MHz, $CD_3OD$) of 3″-hydroxyisosuprine (1).**

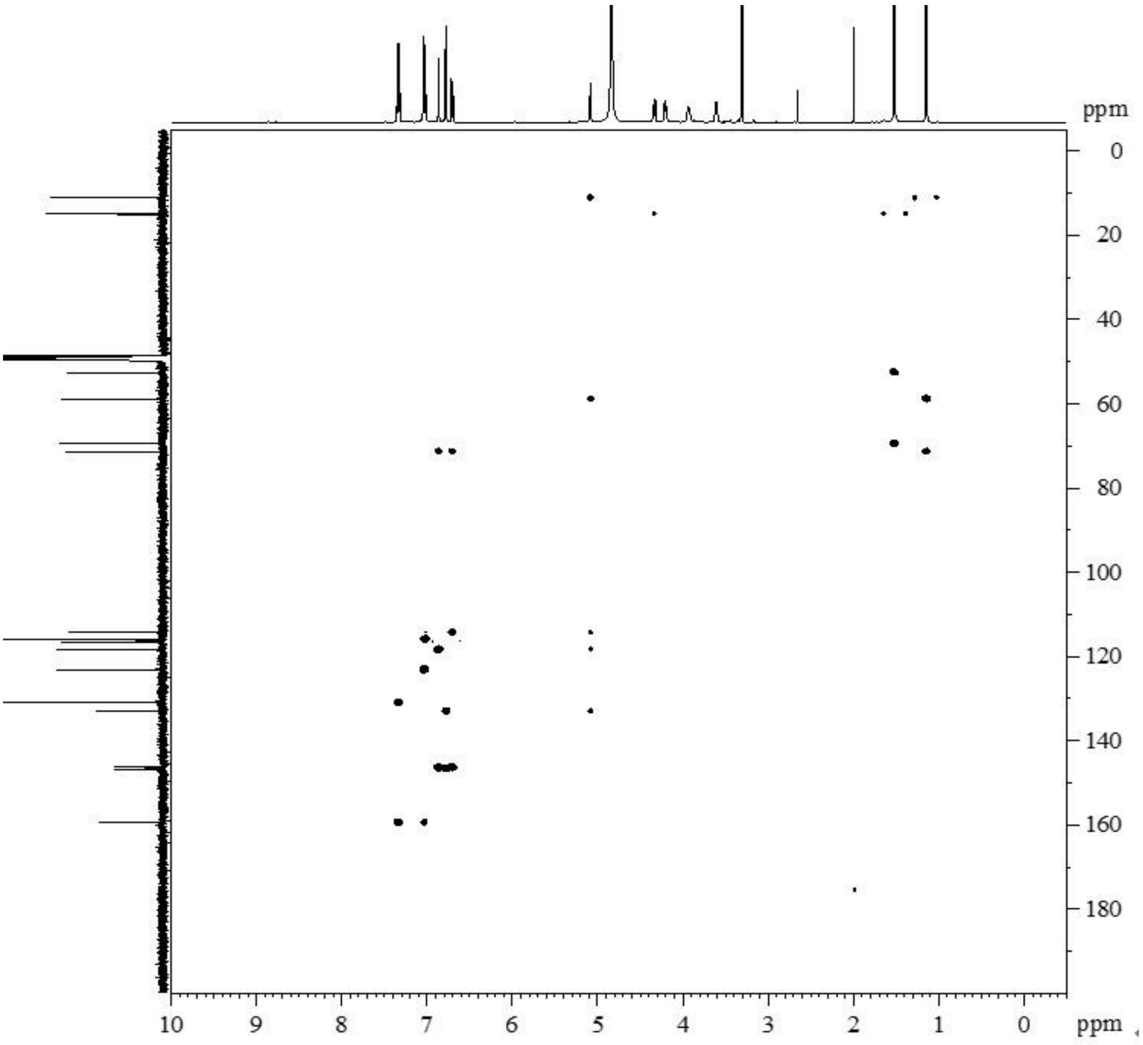


**Fig. S7. 2D NMR spectrum ($^{1}H$-$^{13}C$ HMBC, 500 MHz, py $CD_3OD$) of 3″-hydroxyisosuprine (1).**

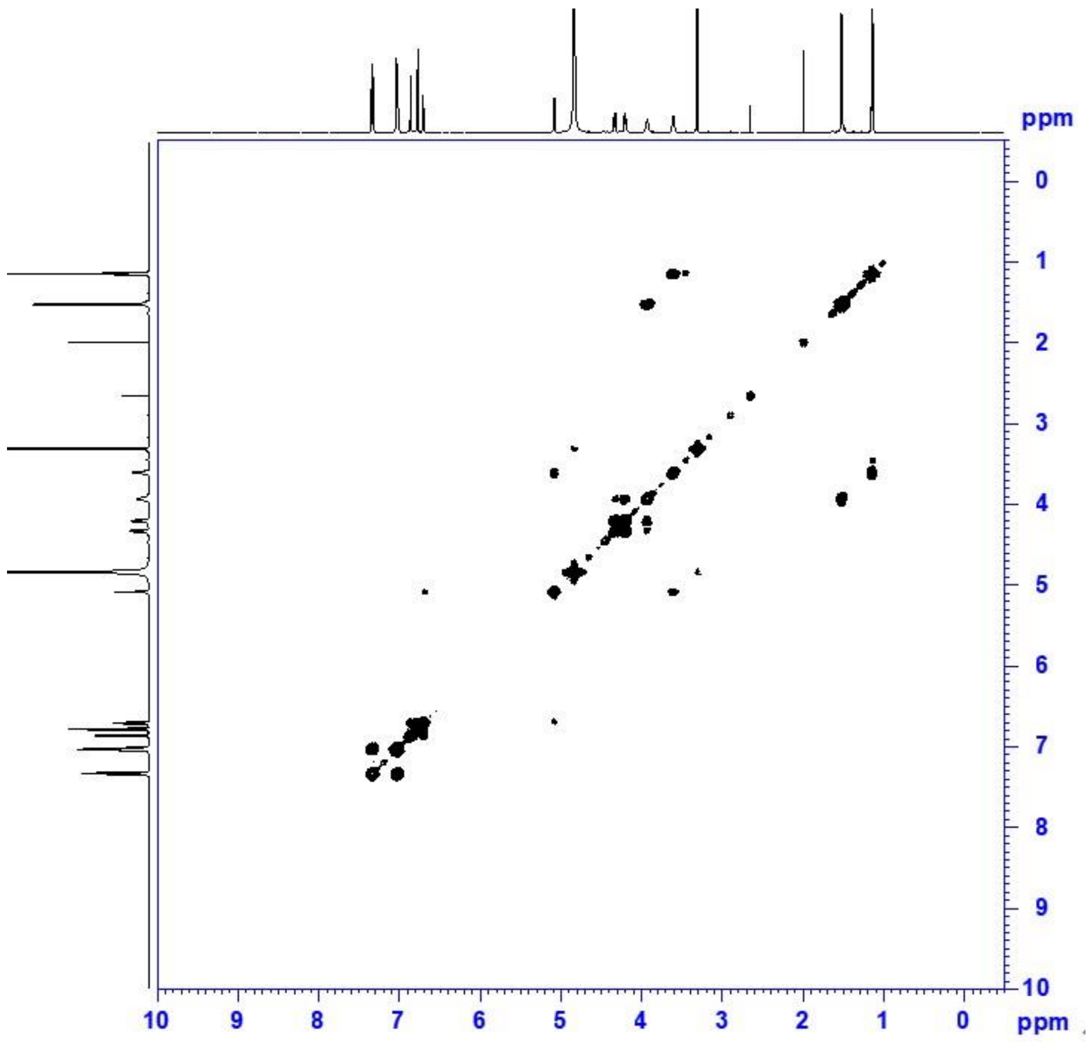


**Fig. S8. 2D NMR spectrum ($^1$H-$^1$H COSY, 500 MHz, $CD_3OD$) of 3″-hydroxyisosuprine (1).**

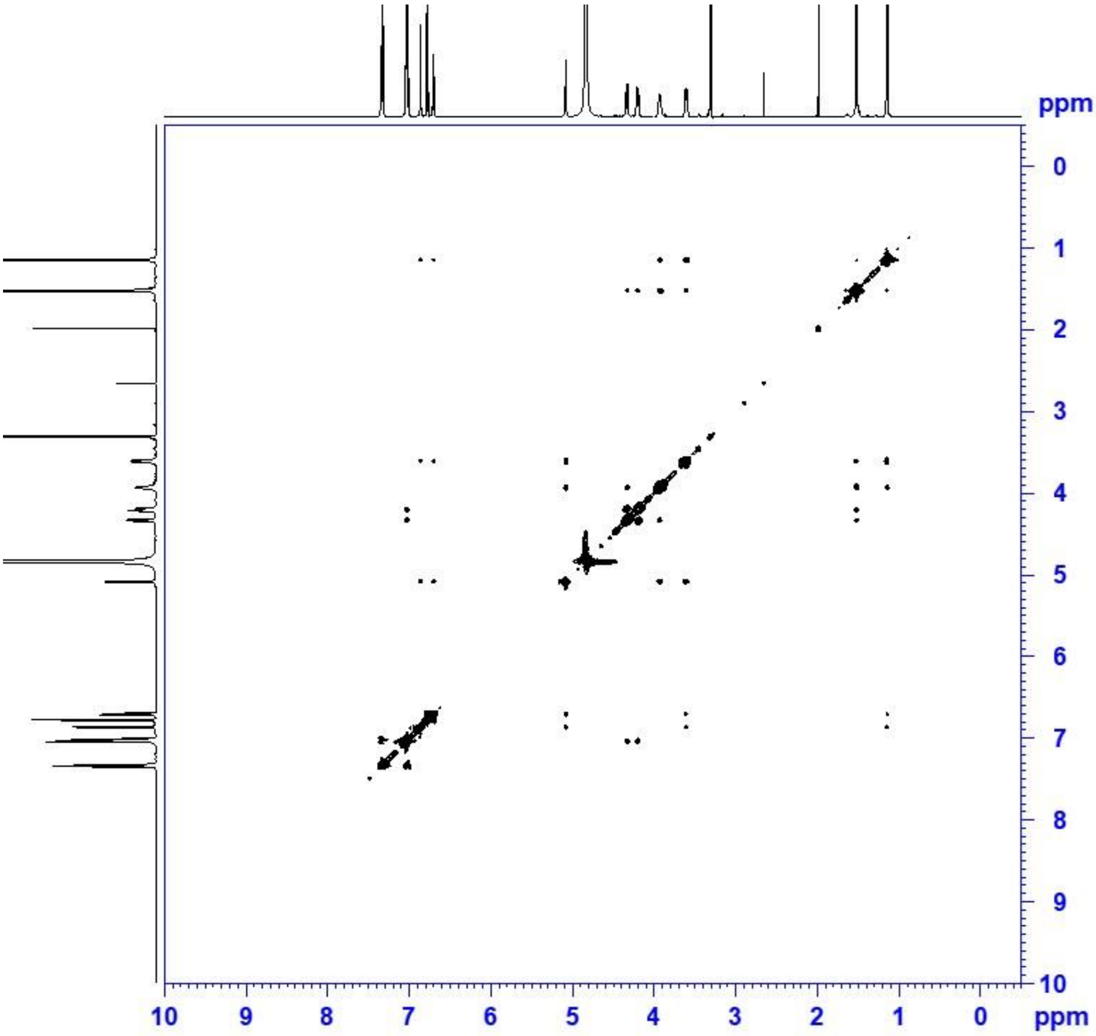


**Fig. S9. 2D NMR spectrum ($^1$H-$^1$H NOESY, 500 MHz, $CD_3OD$) of 3″-hydroxyisosuprine (1).**
